# Examining the performance of responding to the Khoy earthquake 2022, challenges, strengths, and lessons learned: thematic analysis

**DOI:** 10.1186/s13104-024-06845-1

**Published:** 2024-07-02

**Authors:** Saeed Nazari, Pirhossein Kolivand, Elham Zamani, Hamid Karimi Kivi, Razieh Norouzi

**Affiliations:** 1 Health in Disasters and Emergencies, Department of Health in Disasters and Emergencies, AJA University of Medical Sciences, Tehran, Iran; 2 Department of Health Economics, School of Medicine, Shahed University, Tehran, Iran; 3 Research Center for Emergency and Disaster Resilience, Red Crescent Society of the Islamic Republic of Iran, Tehran, Iran; 4 Iran-Helal Applied-Science Higher Education Institute, Red Crescent Society of the Islamic Republic of Iran, Tehran, Iran; 5 Research Center for Health Management in Mass Gathering, Red Crescent Society of the Islamic Republic of Iran, Tehran, Iran

**Keywords:** Earthquake, Lessons learned, Thematic analysis, Khoy

## Abstract

**Objective:**

Disasters in developing countries result in higher human and financial losses compared to global standards, with the death rate being 12 times higher than that of developed countries. Many experts attribute the failures in disaster management to the lack of a system for documenting and analyzing disaster management functions and not leveraging the experiences and lessons learned. This study employed a qualitative data collection approach, utilizing semi-structured interviews with managers, deputies, members of operational teams, and individuals affected by the disaster in the area. This research aims to explore the challenges, strengths, and lessons learned from the response to the Khoy earthquake in Iran.

**Results:**

After conducting 40 interviews and achieving data saturation, we extracted experiences and lessons learned to investigate the performance of responsible organizations in the 2022 Khoy earthquake. The obtained data were categorized into 8 categories and 39 sub-categories. These categories encompassed warning and calling forces, disaster assessment, disaster commanding, emergency housing, supply and distribution of items, organization, and guidance of public participation and charities, psychological support, logistics operations, monitoring, evaluation, documentation, information dissemination, and media management. Planners and operational managers can use the findings to review and revise their action and prevention plans.

## Introduction

According to the Centre for Research on the Epidemiology of Disasters (CRED) [1] a disaster is defined as a “situation or event which overwhelms local capacity, necessitating a request at the national or international level for external assistance; an unforeseen and often sudden event that causes great damage, destruction and human suffering”. The occurrence of various natural and man-made hazards has consistently posed a threat to human life, causing numerous fatalities and injuries while also inflicting substantial economic losses on societies [2–4].

In 2022, there were 387 natural disasters, which is more than the global average of the last two decades (370 cases). The economic damage this year was about 224 billion dollars, compared to the average of the last two decades, which was about 190 billion dollars. This serves as a warning for policymakers and decision-makers in the field of disaster management, urging them to adopt more effective approaches to reduce the effects of disasters [1].

Evidence indicates that, in all countries, efforts to address the exposure of people and infrastructure to disasters have outpaced the reduction of vulnerability. This has led to a continuous increase in the damages caused by disasters, resulting in severe economic, social, health, cultural, and environmental impacts in the short, medium, and long term, particularly at the local and community levels [5].

Iran is one of the most disaster-prone countries in the world, ranking first in terms of the diversity of hazards. Natural disasters in Iran occur frequently and cause significant damages to the affected areas [6]. Out of 43 known types of disasters worldwide, 34 occur in Iran [7]. Additionally, 90% of its population is exposed to natural disasters such as earthquakes and floods [9]. Earthquakes have been the most frequent and destructive natural disasters in Iran, causing over 180,000 deaths in the past 90 years. The 2003 Bam earthquake, with a magnitude of 6.4, is a notable example, resulting in over 30,000 deaths and 10,000 injuries [9].

Many experts argue that the primary cause of failures in disaster management is not human error, but rather the absence of a robust system for documenting and analyzing disaster management functions, and the failure to learn from past experiences and lessons.

This study underscores the significance of coordinated efforts by emergency, health, and treatment organizations in response to the Khoy earthquake on 30th October 2022. The findings outline the challenges, strengths, and lessons learned during the disaster response phase, aiming to improve effectiveness at the scene of the incident. The research identifies the necessary actions, requirements, key skills, and abilities that responsive organizations need in emergency and disaster situations. The study reflects the experiences and lessons learned by responsible organizations during the acute phase of the Khoy earthquake. The insights are based on the experiences of organizations involved in responding to the Khoy earthquake. It is hoped that the findings of this research will be valuable for future planning and implementation by organizations involved in disaster management.

### Methodology

In this qualitative study, an inductive approach was used to conduct semi-structured deep interviews with 27 operational personnel and 13 affected individuals. The participants were selected through targeted and snowball sampling, where each specialist introduced the next specialist, leading to the identification of information-rich individuals. Thematic content analysis was used to analyze the data and identify the challenges and strengths in responding to the Khoy earthquake.

In-depth interviews were carried out with managers, deputies, and operational team members from various organizations involved in the disaster response, including the Red Crescent Society, the Ministry of Health, disaster management agencies, as well as military and security forces operating in the affected area. Additionally, interviews were conducted with individuals impacted by the disaster to gather their perspectives on the performance of the operational teams during field assessments of the affected area. The qualitative data was analyzed using thematic content analysis.

Since the sampling method employed was purposeful snowball sampling, The entry criteria for operational forces included a minimum of 10 years of relevant work experience, as well as field experience and participation in at least 2 disasters. Additionally, willingness to participate in interviews and provide information was required. On the other hand, unwillingness to participate in interviews and limitations in providing sufficient information to the research team were the exclusion criteria.

During the semi-structured interviews, participants were asked to discuss the challenges and strengths of responding to the Khoy earthquake. The experts were asked the following questions:

1. What actions did you take in response to the call and after arriving at the disaster area?
2. How was the operational plan of the dispatched forces?
3. How was the command and management structure of the disaster?
4. How was the security of the area for operations?
5. What was your operational strategy? Why was it decided to distribute items or set up a camp, and on what basis?

Follow-up questions such as “How?”, “Why?”, and “Can you explain more about…?” were also asked during the interviews. A total of 40 interviews were conducted, involving 27 experts and 13 affected individuals. The interviews were concluded once data saturation was achieved, with each interview lasting approximately 45 to 75 min. They were analyzed by a researcher immediately after completion.

The data analysis was carried out at the same time as data collection. Initial codes were assigned to concepts extracted from the items and then categorized into sub-main and main categories. These resulting codes were then grouped into potential categories. The report was analyzed and written by reviewing, defining, and naming subcategories and categories. After the interviews, the texts were analyzed, and codes and categories were categorized. After analyzing the interview texts, removing duplicate codes, and reanalyzing them, 457 codes were extracted, which were classified into 8 categories and 39 subcategories.

To ensure the accuracy of the qualitative data, Guba and Lincoln’s [10] criteria—Credibility, Transformability, Dependability, and Conformability—were used.

## Results

After conducting 40 interviews and reaching data saturation, we extracted experiences and lessons learned from the Khoy earthquake. The data were divided into 8 categories and 39 sub-categories, as shown in Table 1. These categories included warning and calling forces, disaster assessment, disaster commanding, emergency housing, supply and distribution of items, organization and guidance of public participation and charities, psychological support, logistics operations, monitoring, evaluation and documentation, information dissemination, and media management. Furthermore, by examining the response to the Khoy earthquake, we identified the challenges, strengths, and lessons learned in managing this disaster, as shown in Table 2.

**Table 1:** Categories and sub-Categories obtained from interviews with participants

Categories	Sub-categories	Meaning units
Warning and call for forces	Warning and confirmation of occurrence	An online emergency meeting at the Emergency Operation Center (EOC) of the Relief and Rescue Organization with the affected province and certain provinces
	Analysis of available resources	Shortage of operational forces, resources, and equipment in the affected province
	Call for required forces	Calling Operational forces and equipment from certain provinces
Incident evaluation	Initial rapid assessment	Quick evaluation by local teams, rural relief, and rescue teams, and teams stationed at rescue bases near the earthquake center
	Specialized evaluation	Specialized and field evaluation by operational teams in certain provinces
	Data collection and analysis	Uncertainty in data collection and analysis due to multiple aftershocks
	Incident classification	Inappropriate classification of the incident due to inaccurate assessment
	Identification of vital needs	The need for heating equipment and tents due to cold weather and multiple aftershocks
	Continuous evaluation	Continuous evaluation by operational forces due to changes and assumptions
Disaster Commanding	Operational zoning	Division of the affected area into 5 operational zones managed by certain provinces
	Interaction and collaboration between organizations	Weak cooperation of responsible organizations due to lack of personnel
	Incident Command System (ICS)	Lack of unity of command and unified command
	Emergency Response Plan (ERP)	Lack of emergency operation plan and decision-making as required
	Continuous communication during operations	Continuous communication between control and coordination centers for intra-organizational operations
	Safety and security of operational forces	Lack of personal protective equipment during operations
Disaster Commanding	Identification of injured individuals	No accurate and appropriate information and statistics on injured people were available
	Camp standards	Failure to comply with health service standards, bathroom standards, and housing space standards
	Camp safety and security	Safety and security were good in the camp set up in schools and sports halls
Relief item procurement and distribution	Relief item procurement methods	Provision of relief items from public donations, benefactors, and Red Crescent warehouses
	Relief item warehousing	Shortage of storage space in the affected city and use of satellite warehouses
	Relief item distribution methods	The absence of a unified strategy in distributing relief items
	Distribution management and supervision	Poor management and follow-up to deliver items to needy people
	Use of volunteer capacities	Use of volunteer forces’ capacity in distributing items and identifying needy individuals
	Use of local and trusted capacities	Interaction and cooperation with village mayors and councils in distributing items and identifying needy individuals
"Organizing and managing public participation and support."	Attracting, organizing, and directing public aid	Organizational inconsistencies in attracting public aid
	Interaction and collaboration with NGOs	NGOs collect and distribute items separately
	Electronic system information dissemination	Providing account numbers and command codes for cash aid delivery
	Establishment of Mokabs and volunteer stations	Establishment of Mokabs for preparing hot food by volunteer forces in the area
	Public aid campaign creation	Launching the Mehr-e-Taban campaign by the Red Crescent Society for collecting public aid
Psychological support	Identification of needy individuals	The absence of a specific mechanism for identifying needy individuals for psychological support
	Psychological support for adults	Limited psychological support at the city-level camps set up in the affected area
	Psychological support for children (Sahar team)	Creating child-friendly spaces for children’s games
Operational logistics	Transportation fleet management	Inappropriate and suboptimal use of transportation system capacities, especially rail transport
	Relief item procurement management	Participation of 22 provinces in providing necessary relief items
	Human resource management for relief operations	A very large presence of operational forces in the affected area from certain provinces
	Required operational equipment procurement management	Shortage of operational equipment in the logistics section of the affected province, such as lift trucks and unsuitable equipment for operational teams
	Warehouse status in terms of structure and stored items quantity	Failure to comply with storage location standards and inappropriate distribution of items at the provincial level
	Documented statistical report on incoming and outgoing items	Accurate registration of incoming and outgoing shipments and items to warehouses with complete details
	Operational force support (dispatch, nutrition, housing, recovery, psychological support, etc.)	Lack of an appropriate plan for recovery and nutrition of operational forces. The absence of psychological support plans for forces
Monitoring, evaluation, and documentation	Field research and studies	Dispatching field researchers to collect performance information on operational forces
	Review meetings on actions taken	Holding review meetings with operational teams to review actions taken
Information dissemination and media management	Introduction of the single spokesperson	The absence of a single spokesperson, both at headquarters and field level, to present actions taken
	Coordination of news policies with operational command	Increased expectations from affected people due to promises made by some officials that were far from realistic
	Monitoring social media and responding to rumors	Poor management of space and spread of rumors due to multiple aftershocks
	Information dissemination and reporting	Inadequate and documented information dissemination through various media channels

**Table 2:** Challenges, strengths, and lessons learned from responding to the Khoy Earthquake in Iran

Challenges	Uncertainty in data collection and analysis due to changes
	Inadequate coordination and cooperation of the organizations involved in the disaster management process due to the failure to implement the incident command system (ICS) and contingency decisions by managers and officials
	Lack of basic information about the number of people, safe spaces such as schools and parks to set up emergency camps
	Failure to comply with some standards related to the emergency accommodation camp and the distribution of relief items
	Lack of a specific system to identify people in need of accommodation, food items, and psychological support
	The absence of a single spokesperson and the creation of inconsistency in the news and information policies with the operations command
	The existence of specialized operational forces is more than needed in the affected area due to the unstable conditions of the region caused by the occurrence of aftershocks and the lack of a suitable plan for the recovery of these forces
Strengths	Zoning of the affected area into different operational areas and management of these zones by certain provinces
	Establishing emergency accommodation camps in schoolyards and sports halls
	Creating public aid collection campaigns and providing relief items from items donated by people, benefactors, and Red Crescent Society warehouses
	Using the capacity of local educated people, trustees, and volunteers in the initial rapid assessment, setting up the camp, collecting and distributing relief items
	Documented statistical report of the arrival and departure of goods and the existence of real-time statistics of warehouse inventory
	Field research, holding action review meetings with the presence of operational teams, and preparing After Action Report (AAR)
Lessons learned	Handing over the disaster management process in the designated operational area to a certain province and providing the needs and relief items from the same province had provided better management conditions for operations
	The necessity of implementing the incident command system (ICS) and conducting periodic maneuvers and exercises to strengthen the coordination between the forces involved in the disaster management process
	The need to collect basic information related to the population of the region, determine the number of vulnerable groups and the special needs of these groups, and update this information periodically
	Planning and preparation of an operational plan for using the capacity of certain provinces, and practicing the plans developed periodically with the participation of certain provinces
	Planning to attract the active and effective participation of local people, trustees, and volunteers in the process of identifying the victims and distributing relief items
	Decision-making based on the results of initial rapid assessment, even in unstable conditions, is better than decision-making based on guesswork and assumptions. This prevents the wastage of financial, human, and equipment resources

### Warning and calling forces

Following the earthquake in West Azerbaijan Province and its surrounding areas, an emergency online meeting was promptly convened at the EOC of the Relief and Rescue Organization. The Institute of Geophysics, University of Tehran, confirmed the earthquake and relayed the news to the affected province. Notably, the EOC of West Azerbaijan Province successfully established communication with village heads and Red Crescent officials in rural areas to validate public reports and assess initial information.

“As soon as the earthquake struck in East Azarbaijan province, a full alert was issued. Warehouses and operations teams were on high alert.” (P_1_ ).

### Disaster assessment

The rapid disaster assessment was started by sending 5 road bases from West Azerbaijan Province and two bases near the likely epicenter in East Azerbaijan Province (Tabriz and Shabestar). At the same time as the assessment teams were dispatched, information about the disaster's effects was gathered by telephone from Red Crescent officials and village leaders.

“As soon as the earthquake struck, Crescent Home officials were contacted, and their evaluation report was received. Information from the evaluators also confirmed the details provided by Crescent Homes.” (P_2_ ).

Upon arrival at the designated area, all operational teams in surrounding provinces promptly commenced field assessments. However, the results of these assessments were repeatedly disrupted by aftershocks, necessitating reassessment. One of the primary challenges in this area was the occurrence of multiple aftershocks. Over the course of three days following the main earthquake, five aftershocks with a magnitude of approximately 4 Richter or higher altered all collected information and assessments, leading to the need for reassessment.

“Upon arrival in the area (the morning after the earthquake), we conducted a field visit to assess the damage to the area.” (P_5_ ).

### Disaster commanding

As per the directives from the provincial headquarters following the earthquake, Khoy County was promptly divided into 6 operational zones. The neighboring counties were tasked with overseeing the respective zones.

“Shortly after the earthquake, Khoy city was partitioned into 6 districts based on branch proximity. The Urmia, Salmas, Chaldaran, Chaipara, Maku, and Poldasht branches were entrusted with the responsibility for each of these areas.” (P_12_ ).

One of the significant deficiencies in disaster management, unanimously acknowledged by all stakeholders, is the underutilization of the Incident Command System (ICS) and the absence of a unified command structure. Ad hoc decision-making, conflicting orders from multiple individuals at the headquarters, failure of governors to adhere to the chain of command, internal conflicts and disagreements among managers and decision-makers, ambiguous delineation of responsibilities, and disregard for the command structure by city managers and officials have all presented challenges stemming from the lack of ICS implementation.

“The Relief and Rescue Organization failed to follow the established command path outlined in the Incident Command System (ICS). So, it was directly coordinated with provincial relief and rescue deputies and logistics department heads to deploy relief forces, without informing the province's CEO.” (P_28 _).

### Emergency housing

Even though the emergency housing operations for destroyed homes and those in need of repair ended at 8 a.m. the day after the disaster, most people whose homes were damaged were provided with emergency housing. However, participants highlighted that emergency housing posed the main challenge in managing this earthquake. While some individuals mentioned cold weather, cultural issues, and multiple aftershocks, more participants and experts pointed to inappropriate initial assessment and the lack of a unified and appropriate strategy in emergency housing. Additionally, the parallel work of multiple organizations and institutions in distributing items, especially relief tents, was cited as a major reason for these challenges.

The strategy of establishing camps in school yards, adopted by some neighboring provinces in their operational areas, proved to be a key strength in disaster management. The selection of enclosed locations such as schoolyards and sports halls was particularly advantageous. These sites were well-suited for setting up camps, providing a secure space for operational teams and families, facilitating item distribution and follow-up, and offering infrastructure such as sanitation facilities.

“One advantage of setting up camps in school yards was that people were allowed in with their national ID cards, and this allowed for better record-keeping of all individuals. Additionally, if individuals wanted to leave, they could simply evacuate the tent and depart. In parks and open spaces, people would take their tents with them when leaving. Furthermore, setting up camps reflected the effectiveness of organizations’ operation, especially in situations where it was not clear what would happen to the items distributed among individuals in cities and villages.” (P_35 _).

### Relief item procurement and distribution

In the distribution of items, a consistent strategy was lacking, resulting in the use of various methods of direct and indirect, centralized and decentralized distribution. Due to inadequate initial assessment, the distribution of tents and relief items was irregular and inappropriate. It lacked coordination and uniformity, with each organization and institution distributing based on their own data. Consequently, in many selected areas for distribution, relief items and tents were distributed by other organizations either before or after.

“The allocation of resources commenced with the rural councils and subsequently transitioned to the Red Crescent. However, discrepancies in the data provided by the rural councils resulted in challenges pertaining to the accurate distribution and delivery of the resources to the intended recipients.” (P_22 _).

### Organizing and managing public participation and charitable activities

One commendable action that was widely praised by participants was the initiation of the Mehr-e-Taban campaign to gather public cash donations. This campaign facilitated the collection of cash donations through four methods: central bank account number, card number, and command code, as well as through collection boxes placed in city-level stations.

### Psychological support

Due to ongoing aftershocks and widespread rumors causing increased public fear and collective stress, participants emphasized the importance of establishing child-friendly spaces in camps during discussions on psychological support. However, the limited availability of personnel and equipment restricted these activities to camps only, leaving other affected individuals without the psychological support they also needed.

“We implemented programs in approximately 25 camps. Revisiting most of the camps was necessary; however, due to the shortage of staff, this was not possible.” (p _17 _).

### Logistics of operations

The support for operational forces was executed through various methods. In certain provincial areas, activities such as dispatching and supporting, sending supplies, and providing food and accommodation for the forces were coordinated. The allocation and distribution of relief items were organized at the provincial level and across regions, however, there was a lack of effective information sharing between units. Additionally, many participants highlighted the absence of a plan to source essential items from local resources as a significant concern.

“Local resources such as mineral water and bread, abundant in the center of the province, were not utilized; instead, mineral water was transported from distant provinces using compression.” (P_26 _).

### Information dissemination and media management

As there was no spokesperson for the command unit to communicate the actions taken and information related to disaster management, and no commander for the operational unit, multiple officials spoke with the media. Both individuals and officials raised various issues that were not aligned with disaster management and command. In some cases, they raised the expectations of disaster victims by making promises that were far from realistic.

## Discussion

Effective disaster response is a significant challenge for governments worldwide. A centralized governance system is not conducive to effective disaster response [11].

The study's findings indicate that the initial step in deciding whether to call first responder teams involves engaging with local individuals, particularly officials of Hilal Houses in various earthquake-affected areas. The involvement of these local figures is pivotal in swiftly assessing and reporting the situation for the preliminary call for assistance. Based on the outcomes of the initial rapid assessment, it becomes feasible to identify the necessary operational teams and mobilize them. To effectively manage the response operation, comprehensive research into the disaster location and its actual conditions is imperative. Seamless coordination at all levels is crucial, and there should be premeditated solutions for managing the response prior to, during, and after the disaster for each involved organization [12, 13]. As discussed in the findings section, the occurrence of multiple aftershocks not only disrupted the evaluation results but also led to heightened public panic. In such circumstances, the presence of volunteers and non-governmental organizations can be invaluable. However, it's crucial to emphasize that self-initiated organizations, without proper coordination, can inadvertently exacerbate the situation. For instance, following the devastating earthquake in Haiti, the influx of support from the US health community resulted in many well-intentioned organizations offering their assistance. Unfortunately, due to a lack of coordination, their skills couldn’t be immediately utilized in an effective manner [14].

Support and assistance should be provided in proportion to the population density and vulnerability of the affected area. Inadequate logistics could lead to loss of life among vulnerable and injured groups due to medical limitations [15, 16]. Furthermore, coordination at the neighborhood level among individuals, stakeholders, local organizations, municipalities, and community centers, as well as at the community level between local authorities, organizations, and government entities such as ministries, is vital for effective disaster management [17].

Disaster management entails the coordination of public and private institutions, volunteer groups, affected communities, and the media. Information, or the absence of it, can significantly influence all stages of a disaster. As such, the media and the presence of a designated spokesperson are essential for effective communication and comprehension of disasters and their aftermath [18, 19].

## Conclusion

This research examined the response to the Khoy earthquake, highlighting the challenges, strengths, and lessons learned in managing this disaster. It identified the lack of unified command, contingency planning, and difficulties in emergency accommodation and item distribution. The study emphasized the importance of pre-disaster planning and the potential impact of aftershocks on data collection and emergency management. The research recommended comprehensive emergency management planning, especially in vulnerable areas, as well as the need for specific plans for emergency accommodation, relief item distribution, and public information. The findings can serve as a basis for updating disaster response plans, policies, and procedures in Iran, helping operational managers and planners to revise and rewrite their plans based on the study results.

### Limitation

The limitation of the study was the lack of access to the managers of the organizations responding to the earthquake during the emergency management of the disaster. Although the researchers tried to ask the opinions of the commanders and managers about the research questions after the emergency subsided. However, it is possible that these opinions and information that they provided may have undergone changes after a few days of the disaster.

## Data Availability

The datasets generated and/or analyzed during the current study are available from the corresponding author upon request.

## Abbreviations

CRED: The Centre for Research on the Epidemiology of Disasters
EOC: Emergency Operation Center
ICS: Incident Command System
ERP: Emergency Response Plan
NGOs: Non-Government Originations
AAR: After Action Report
CEO: Chief Executive Officer
